# The Impact of Vision Loss on Allocentric Spatial Coding

**DOI:** 10.3389/fnins.2020.00565

**Published:** 2020-06-16

**Authors:** Chiara Martolini, Giulia Cappagli, Antonella Luparia, Sabrina Signorini, Monica Gori

**Affiliations:** ^1^Unit for Visually Impaired People, Istituto Italiano di Tecnologia, Genoa, Italy; ^2^Department of Informatics, Bioengineering, Robotics and Systems Engineering, University of Genoa, Genoa, Italy; ^3^Center of Child Neuro-Ophthalmology, IRCCS Mondino Foundation, Pavia, Italy

**Keywords:** visual impairment, spatial frame of reference, allocentric reference frame, egocentric reference frame, spatial perception

## Abstract

Several works have demonstrated that visual experience plays a critical role in the development of allocentric spatial coding. Indeed, while children with a typical development start to code space by relying on allocentric landmarks from the first year of life, blind children remain anchored to an egocentric perspective until late adolescence. Nonetheless, little is known about when and how visually impaired children acquire the ability to switch from an egocentric to an allocentric frame of reference across childhood. This work aims to investigate whether visual experience is necessary to shift from bodily to external frames of reference. Children with visual impairment and normally sighted controls between 4 and 9 years of age were asked to solve a visual switching-perspective task requiring them to assume an egocentric or an allocentric perspective depending on the task condition. We hypothesize that, if visual experience is necessary for allocentric spatial coding, then visually impaired children would have been impaired to switch from egocentric to allocentric perspectives. Results support this hypothesis, confirming a developmental delay in the ability to update spatial coordinates in visually impaired children. It suggests a pivotal role of vision in shaping allocentric spatial coding across development.

## Introduction

The ability to locate targets in the environment is a critical aspect of spatial information processing, and consequently, it has been extensively considered a milestone for space development (Lew et al., 2000; Vasilyeva and Lourenco, 2012; Cappagli and Gori, 2019). The cognitive representation of space is given by the reciprocal relationships between entities in the environment. It is strongly dependent on the perspective assumed by the perceiver, namely the frame of reference that allows us to keep track of and continuously update objects’ position in space. While the *egocentric* or *subject-centered* perspective references spatial objects’ locations to the perceiver’s own body, the *allocentric* or *object-centered* frame of reference refers to objects’ locations based on external landmarks, such as objects other than the body (Klatzky, 1998; Foley et al., 2015).

Empirical evidence suggests that allocentric spatial coding is promoted by the ability to combine perceptual experiences of an environment (Nardini et al., 2009; Vasilyeva and Lourenco, 2012) and by visual experience across development (Thinus-Blanc and Gaunet, 1997; Pasqualotto et al., 2013). Understanding the context in which egocentric instead of allocentric frames of reference need to be chosen (and vice-versa) depends on the capacity to integrate different spatial coordinates systems to encode space. Research has shown that adults typically employ a spatial strategy based on the integration of egocentric and allocentric frames of reference [e.g., Nadel and Hardt (2004)]. Contrarily, children start to integrate the two reference frames only around 6 years of age (Nardini et al., 2006; Bullens et al., 2010); see also (Nardini et al., 2008). This result is evident even if egocentric and allocentric representations coexist already before that age (Newcombe and Huttenlocher, 2003; Nardini et al., 2006). Indeed, it has been shown that typically developing children attempt to locate objects using allocentric landmarks within 1 year of age. While from 8.5 months of age they rely on adjacent landmarks (cue learning) to find non-visible targets (Piaget and Inhelder, 1967; Acredolo, 1981), they start to rely on distal landmarks (place learning) at the age of 12 months (Lew et al., 2000). This finding suggests a developmental acquisition of allocentric capabilities. At 24 months of age, toddlers show the ability to rely on distal cues (Newcombe et al., 1998), consolidating the consciousness of relations between distal landmarks throughout childhood (Rieser and Rider, 1991; Overman et al., 1996; Nardini et al., 2009; Vasilyeva and Lourenco, 2012). Nonetheless, switching-perspective abilities rely on the capacity to efficiently integrate egocentric and allocentric frames of reference, which is still not mature until 8 years of age (Nardini et al., 2008).

Several studies have demonstrated that vision plays a relevant role in the acquisition of spatial knowledge. Indeed, vision not only permits to perceive multiple stimuli at the same time (Foulke, 1982; Thinus-Blanc and Gaunet, 1997; Merabet and Pascual-Leone, 2010; Pasqualotto et al., 2013; Iachini et al., 2014), but it also allows to acquire the ability to code spatial information in allocentric coordinates. At the cortical level, visual experience seems to shape the architecture of cortical spatial maps by providing the most accurate spatial information (Maurer et al., 2005; Lepore et al., 2009; Ruotolo et al., 2012). As a consequence, visual loss may significantly affect an adequate spatial representation of the external world (Ungar et al., 1995; Bigelow, 1996; Cattaneo et al., 2008; Koustriava and Papadopoulos, 2010). In line with this view, it has been demonstrated that visually impaired adults tend to code space mainly through an egocentric perspective, probably because they rely on sensory modalities other than vision, that is based on body landmarks (i.e., touch; Cattaneo et al., 2008; Pasqualotto et al., 2013). Furthermore, the absence of vision prevents the ability to solve spatial tasks that require the use of allocentric cues (Millar, 1994; Thinus-Blanc and Gaunet, 1997; Cattaneo et al., 2008; Merabet and Pascual-Leone, 2010; Pasqualotto and Proulx, 2012; Schmidt et al., 2013; Iachini et al., 2014). It also impacts on the ability to update flexibly and combine different (egocentric/allocentric) reference frames in response to environmental changes – i.e., switching-perspective skills (Cornoldi et al., 1991; Nadel and Hardt, 2004; Vecchi et al., 2004; Burgess, 2006; Harris et al., 2012). Ruggiero et al. (2018), for instance, evaluated how congenitally blind adults performed a switching-perspective task based on the memorization of haptic spatial stimuli. Their work demonstrated that congenitally blind individuals showed relevant difficulties in switching from external (allocentric) to body-centered (egocentric) frames of reference, but not vice-versa. These findings suggest that normally sighted and individuals with a visual impairment might differently encode spatial information from an early age, especially when visual deprivation negatively impacts on multisensory integration capabilities, upon which spatial competence is based (Thinus-Blanc and Gaunet, 1997; Cappagli et al., 2015; Vercillo et al., 2016). Nonetheless, very little is known about the development of switching-perspective abilities in visually impaired children. Ochaíta and Huertas (1993) suggest that normally sighted and visually impaired children acquire a coherent sense of space, respectively, at 14 and 17 years of age. This can be explained by the fact that visual deprivation results in a lack of sensorimotor (visuo-motor) feedback that delays locomotor development (Fraiberg, 1977; Landau et al., 1984; Fazzi et al., 2002), which has been indicated as a fundamental step for spatial competence development (Bremner et al., 2008). Similarly, other studies indicate that, along with locomotor delays, visually impaired children manifest deficits in performing mental spatial tasks, as mental rotations of the self (perspective-taking; Huttenlocher and Presson, 1973; Millar, 1976; Papadopoulos and Koustriava, 2011; Koustriava and Papadopoulos, 2012), or objects/configurations (Huttenlocher and Presson, 1973; Penrod and Petrosko, 2003; Papadopoulos and Koustriava, 2011). Understanding whether and how visually impaired children develop switching-perspective abilities would be fundamental to increase knowledge about the role of vision in spatial development. Indeed, to date it is still unclear whether the complete (such in the case of blindness), or the partial (such in the case of visual impairment) loss of vision would differently affect the ability of children to acquire an allocentric coding of space.

In the present work, we assessed whether the ability to switch from egocentric to allocentric coordinates is compromised by a partial loss of vision that produces an impoverished visual experience during childhood. We hypothesized that children with an atypical visual experience during development (visually impaired) would rely more heavily on egocentric coordinates and, thus, would show an impairment in task conditions requiring a mental update of spatial configurations according to their new perspective.

To test our hypothesis, we assessed the ability of children with typical and atypical visual experience to switch from an egocentric to an allocentric representation of space in the visual domain. Both visually impaired and normally sighted participants were asked to reproduce a spatial configuration of visual stimuli in four conditions that differed in their reliance on visual input, to understand if vision is crucial to acquire switching-perspective abilities. More specifically, participants were asked to change their physical position in space and mentally rotate the spatial configuration seen according to their new coordinate system to accomplish the task.

## Materials and Methods

### Participants

Normally sighted and visually impaired children between four and nine years of age were enrolled in the study. Normally sighted children were recruited from local schools, visually impaired children were recruited from a local hospital (IRCCS Mondino Foundation, Pavia, Italy) based on their visual acuity (VA). The visual deficit was defined through specific tests following the “International Statistical Classification of Diseases and Related Health Problems” [ICD-10 (World Health Organization [WHO], 1993)], which defines moderate to severe visual impairment as a condition characterized by VA comprised between 0.5 and 1.3 LogMAR [Logarithm of the Minimum Angle of Resolution, defined as *log*_10_(*M*_inimum_*A*_ngle_*R*_esolution_)]. Only visually impaired children presenting with best corrected binocular VA in the range 0.5–1.3 LogMAR were recruited (see Table 1 for clinical details of participants). All distance VA measurements were carried out at a testing distance of 3 m. To control for cognitive development, visually impaired children undertook the verbal scale of the “Wechsler Preschool and Primary Scale of Intelligence” (Wechsler, 2012), and the “Wechsler Intelligence Scale for Children” (Wechsler, 2014) according to their chronological age. Only children presenting adequate cognitive development were recruited. Neither visually impaired nor normally sighted children reported additional sensory, musculoskeletal, neurological disabilities, or impairments related to colors discrimination. All normally sighted children had binocular best corrected VA of 0.0 LoGMAR or better. Twenty-seven normally sighted (mean age: 6.56 ± 1.80 years) and fifteen visually impaired (mean age: 6.33 ± 1.72 years) children participated in the study. Both visually impaired and normally sighted participants were divided into three groups, according to their age range: 4-to-5 years old (five visually impaired, nine normally sighted), 6-to-7 years old (six visually impaired, ten normally sighted), and 8-to-9 years old (four visually impaired, eight normally sighted). The study was approved by the local Ethical Committee and written informed consent was provided by participants’ parents, in accordance with the Declaration of Helsinki.

**TABLE 1:** Clinical details of visually impaired participants.

Participant	Age range	Pathology	Visual Acuity (LogMAR)
#1	4–5	Left Micropthalmia and Bilateral Coloboma	1.00
#2	4–5	Nystagmus	1.00
#3	4–5	Retinal Dystrophy	1.00
#4	6–7	Retinopathy	1.30
#5	8–9	Microphtalmia	1.00
#6	6–7	Aniridia	1.30
#7	6–7	Nystagmus	1.00
#8	8–9	Retinal Dystrophy	1.00
#9	4–5	Albinism	0.82
#10	6–7	Bilateral Micropthalmia and Coloboma	1.30
#11	4–5	Optic Nerve Hypoplasia	1.30
#12	8–9	Retinal Dystrophy	0.82
#13	6–7	Nystagmus	0.50
#14	8–9	Optic Nerve Hypoplasia	1.00
#15	6–7	Retinopathy	1.00

*The table shows the age range at test, the pathology, and Visual Acuity (VA) expressed in LogMAR scale at a distance of 3 m of visually impaired participants.*

### Procedure

**FIGURE 1:**
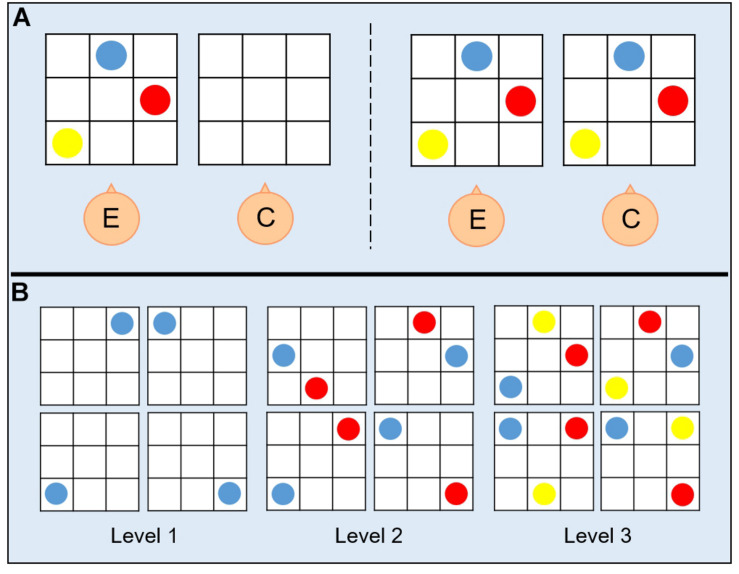
**(A)** Procedure for the switching-perspective task. On each trial, the experimenter (E) showed to the child (C) one out of twelve possible configurations of colored coins on a plastic board made of nine boxes (left panel) and immediately after the child was asked to reproduce the same configuration on his own plastic board in front of him (right panel). **(B)** Trials for each condition of the task. The switching-perspective task comprised 48 trials that differ with respect to the level of difficulty, namely to the number of coins that constituted the configuration to be reproduced (from one to three coins, respectively, for the easiest and the hardest levels).

**FIGURE 2:**
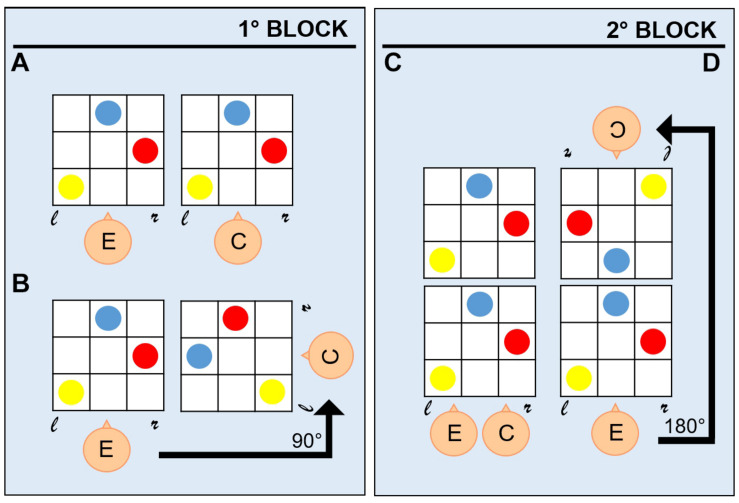
Egocentric and allocentric conditions. Four conditions were administered to participants in two separate blocks: each block comprised both an egocentric **(A,C)** and an allocentric **(B,D)** condition. In the first block (left panel), the allocentric condition **(B)** resulted from a 90° rotation respect to the egocentric condition **(A)**. In the second block (right panel), the allocentric condition **(D)** resulted from a 180° rotation respect to the egocentric condition **(C)**. *E: experimenter; C: child; *r*: right; and *l*: left.

**FIGURE 3:**
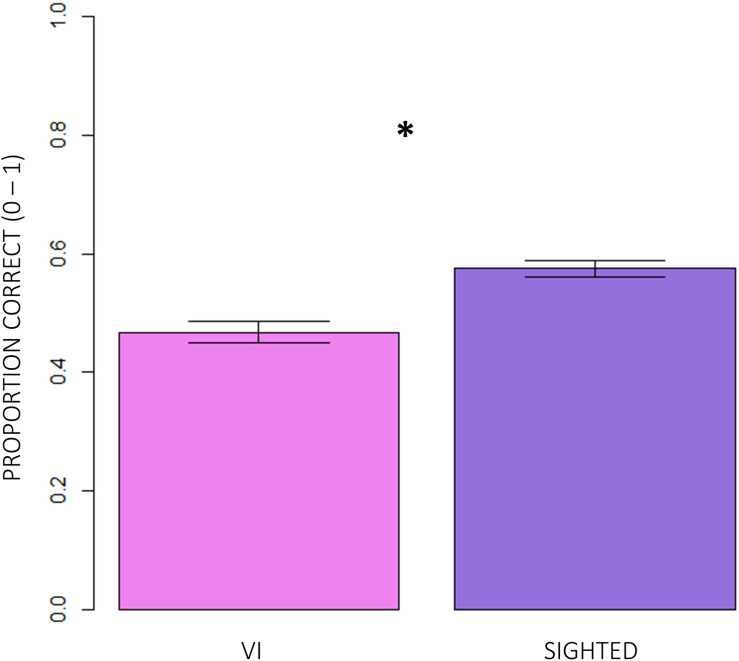
Comparison of correctness of responses between visually impaired and normally sighted participants. The inter-groups analysis showed that visually impaired (VI) children significantly performed significantly worse than normally sighted children (RSS = 5.13, iter = 5000, *p* < 2e–16; and Bonferroni: *p* = 0.00), independently of the age group participants belonged to and of the difficulty of conditions.

**FIGURE 4:**
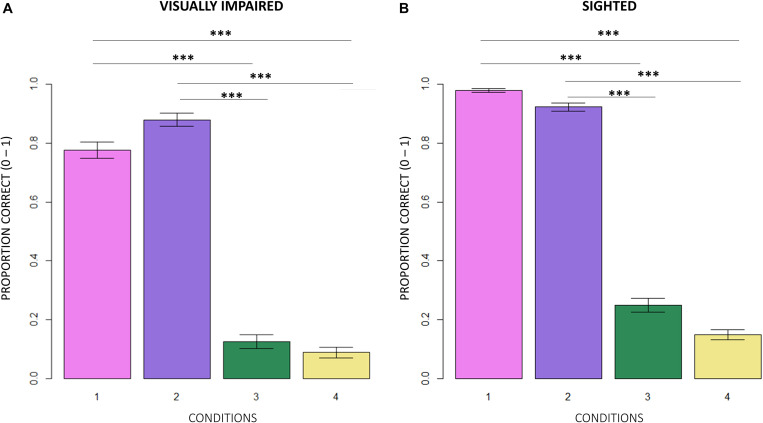
Intra-group comparison between conditions. **(A)** In egocentric conditions (1, 2), visually impaired participants scored significantly higher in correct responses than in allocentric conditions (3, 4; RSS = 71.50, iter = 5000, *p* < 2e–16; and Bonferroni: 1 vs. 3 = 1 vs. 4 = 2 vs. 3 = 2 vs. 4: *p* = 0.00). **(B)** Normally sighted participants obtained a similar result in egocentric conditions (RSS = 162.45, iter = 5000, *p* < 2e–16; and Bonferroni: 1 vs. 3 = 1 vs. 4 = 2 vs. 3 = 2 vs. 4: *p* = 0.00).

A switching-perspective task was administered to all participants. Participants sat in the experimental room with the setup positioned in front of them on a table. The setup consisted of two 30 × 30 cm plastic boards, whose layout represented a grid with intersecting embossed straight vertical and horizontal lines used to separate boxes, on which colored coins (red, blue, and yellow) were positioned (Figure 1A). The boards were realized in such a way that children with visual impairment could visually discriminate stimuli by relying on high contrast colors (colored coins on high contrast background). Before the beginning of the task, the experimenter showed the participant a configuration with an increasing number of coins to let the child familiarize with the task. The task procedure comprised two phases: (a) a demonstration phase, during which the experimenter asked the participant sitting next to him/her to look at a configuration presented on the experimenter’s board; (b) a reproduction phase, during which the participant was asked to reproduce on his board the configuration of the coins shown in (a) by assuming one out of four different spatial positions. During both phases, participants were allowed to look at the experimenter’s configuration as many times as they needed to reproduce it. Depending on the number of coins in the configuration presented by the experimenter in the demonstration phase (a), the task assumed three levels of difficulty (Figure 1B): (1) one coin, for the simplest level; (2) two coins, for the intermediate level; and (3) three coins, for the hardest level. Configurations were presented to the participant in random order concerning the level of difficulty. The participant could assume four spatial positions during the reproduction phase (b), which defined the four conditions of the switching-perspective task: (1) egocentric condition, with the participant sitting next to the experimenter (0° rotation degrees) and the two boards lying next to each other (Figure 2A); (2) egocentric condition, with the participant sitting next to the experimenter (0° rotation degrees) and the two boards lying one above the other (Figure 2C); (3) allocentric condition, with the participant sitting rotated 90° degrees to the experimenter position and the boards positioned in front of them (Figure 2B); (4) allocentric condition, with the participant sitting rotated 180° degrees to the experimenter position and the boards positioned in front of them (Figure 2D). The four different spatial conditions defined two reference frames (egocentric vs. allocentric). The main goal of such manipulation was to asses the participants’ ability to switch from an egocentric to an allocentric frame of reference. The task procedure comprised two blocks of trials. To randomize the presentation of egocentric and allocentric spatial positions, each block comprised one configuration with participants sat next to the experimenter and one configuration with participants sat rotated to the experimenter. The first block (Figure 2, left panel) comprised trials with positions (1) and (3), the second block (Figure 2, right panel) included trials with positions (2) and (4). The total amount of trials performed by each participant was 48 (twelve trials per four spatial positions, four trials for each level of difficulty). The whole experiment was performed on the same day in about 1 h, and short breaks were allowed at any time during the session.

### Data Analysis and Statistics

The accuracy in the task was measured to quantify the spatial ability to switch from an egocentric to an allocentric frame of reference in children with and without visual impairment. We computed a correctness score, as follows:

(1) $$CS=\frac{\sum_{i=1}^{N}ncr}{N},$$

where *CS* stands for “Correctness Score,” *ncr* stands for the *n*umber of *c*orrect *r*esponses for each of the four task conditions defined by the position assumed by the participant during the reproduction phase (egocentric – 1, egocentric – 2, allocentric – 3, allocentric – 4, and see Figure 2), and *N* stands for the number of repetitions per condition (12 trials). Responses were considered as correct (trial score = 1) when the participant accurately reproduced the configuration presented by the experimenter during the demonstration phase, despite the spatial position assumed during the reproduction phase and thus despite the confounding visual feedback of the whole scene. For instance, while in the egocentric conditions children can rely on the visual feedback of the scene to copy the layout configuration, in the allocentric conditions they had to mentally rotate the board layout (90° in condition 3, 180° in condition 4) to place the coins correctly according to the configuration presented (see a comparison of conditions 1/2 and 3/4 in Figure 2). Therefore, correct responses for the two egocentric conditions (Figures 2A,C) were considered as egocentric responses because correct reproduction was based on egocentric coordinates, while correct answers for the two allocentric conditions (Figures 2B,D) were considered as allocentric responses because correct reproduction was based on the ability to switch from an egocentric to an allocentric frame of reference. Moreover, we computed a score for “specular” and “casual” responses, given when children positioned coins in a mirror-like configuration with respect to the assumed midline and in a way that could not be linked to any of the categories mentioned above, respectively. We evaluated the normal distribution of data applying the Shapiro–Wilk test of normality with the free software R (Free Software Foundation, Boston, MA, United States). Since we verified that data did not follow a normal distribution, we used non-parametric methods for the analysis. Two levels of analysis were performed: an intra-group level, which considered the performance of visually impaired and normally sighted children to investigate developmental trends separately; an inter-groups level, which compared the performance of visually impaired and normally sighted participants. Starting from the intra-group level, we conducted four separate mixed permuted ANOVAs with “*correct*,” “*specular*,” “*egocentric*” (only allocentric conditions), and “*casual*” responses as dependent variables, within-factors “*age groups*” (three levels: 4–5, 6–7, and 8–9), “*coins*” (three levels: One, Two, and Three), and “*conditions*” (four levels: 1, 2, 3, and 4) as independent variables. For the inter-groups level, we performed four separate mixed permuted ANOVAs with “*correct*,” “*specular*,” “*egocentric*” (only allocentric conditions), and “*casual*” responses as dependent variables, between-factor “*subjects*” (two levels: *Visually Impaired*, Normally Sighted), and within-factors “*age groups*” (three levels: 4–5, 6–7, and 8–9), “*coins*” (three levels: One, Two, and Three), and “*conditions*” (four levels: 1, 2, 3, and 4) as independent variables. The permuted Bonferroni correction for non-parametric data was applied in case of significant effects to adjust the *p*-value of multiple comparisons (significant value: *p* < 0.05).

## Results

**FIGURE 5:**
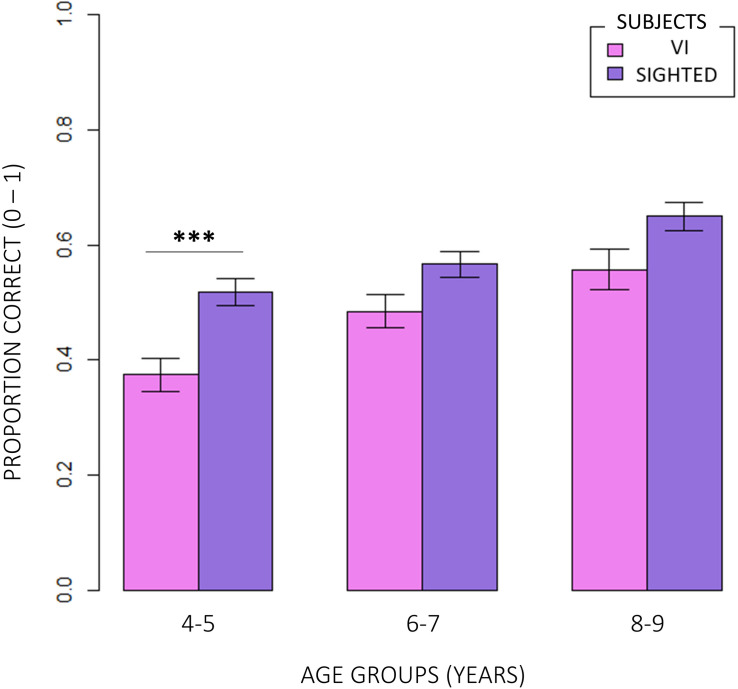
Inter-groups comparison between age groups. Visually impaired participants performed significantly worse compared to normally sighted peers at 4–5 than 6–7 and 8–9 years of age (RSS = 0.21, iter = 5000, *p* = 0.0012; and Bonferroni: 4–5VI vs. 4–5S: *p* = 0.00; 6–7VI vs. 6–7S: *p* = 0.06; 8–9VI vs. 8–9S: *p* = 0.10).

**FIGURE 6:**
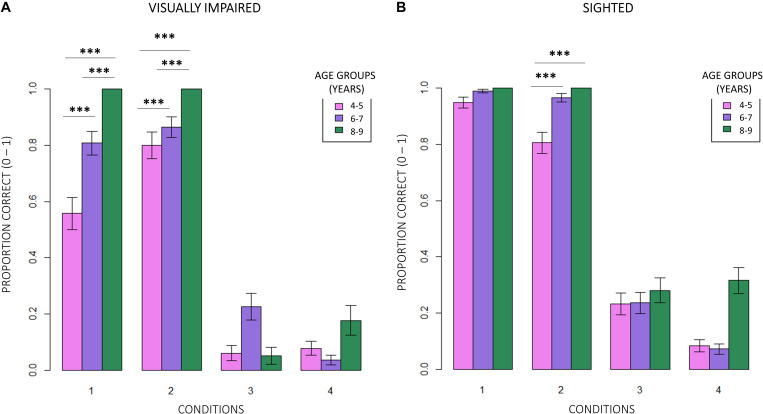
Intra-group evaluation of accuracy among egocentric conditions. **(A)** Visually impaired children showed a strong developmental trend (RSS = 2.09, iter = 5000, *p* < 2e–16; and Bonferroni: 4–5_1 vs. 6–7_1: *p* = 0.00; 4–5_1 vs. 8–9_1: *p* = 0.00; 6–7_1 vs. 8–9_1: *p* = 0.00; 4–5_2 vs. 8–9_2: *p* = 0.00; and 6–7_2vs. 8–9_2: *p* = 0.00). **(B)** On the contrary, only 4–5 years old normally sighted participants showed a similar trend, and exclusively in condition 2 (interaction between age groups × conditions; RSS = 0.35, iter = 5000, *p* < 2e–16; and Bonferroni: 4–5_2 vs. 6–7_2: *p* = 0.00; 4–5_2 vs. 8–9_2: *p* = 0.00).

Firstly, we compared the correctness of responses between normally sighted and visually impaired participants. Figure 3 shows that, independent of the age group participants belonged to and of the difficulty of conditions, visually impaired (VI) children significantly reported less correct responses than normally sighted peers (inter-groups analysis; main effect: subjects; RSS = 5.13, iter = 5000, *p* < 2e–16; and Bonferroni: *p* = 0.00). The intra-group analysis underlined significant differences between conditions (Figure 4). In egocentric conditions (1, 2), correct responses were significantly higher than in allocentric conditions (3, 4) for both normally sighted (main effect: conditions; RSS = 162.45, iter = 5000, *p* < 2e–16; and Bonferroni: 1 vs. 3 = 1 vs. 4 = 2 vs. 3 = 2 vs. 4: *p* = 0.00), and visually impaired (main effect: conditions; RSS = 71.50, iter = 5000, *p* < 2e–16; and Bonferroni: 1 vs. 3 = 1 vs. 4 = 2 vs. 3 = 2 vs. 4: *p* = 0.00) participants. Moreover, the different effect size between normally sighted and visually impaired children seemed to be related to a development factor (inter-groups analysis; interaction between subjects *x* age groups; RSS = 0.21, iter = 5000, and *p* = 0.0012). In Figure 5, visually impaired participants performed significantly worse compared to normally sighted peers at 4–5 than 6–7 and 8–9 years of age (Bonferroni: 4–5VI vs. 4–5S: *p* = 0.00; 6–7VI vs. 6–7S: *p* = 0.06; 8–9VI vs. 8–9S: *p* = 0.10). Starting from these findings, a deeper evaluation of accuracy among egocentric conditions revealed a dependency on age groups (Figure 6). Indeed, visually impaired children showed a strong developmental trend (interaction between age groups × conditions; RSS = 2.09, iter = 5000, *p* < 2e–16; and Bonferroni: 4–5_1 vs. 6–7_1: *p* = 0.00; 4–5_1 vs. 8–9_1: *p* = 0.00; 6–7_1 vs. 8–9_1: *p* = 0.00; 4–5_2 vs. 8–9_2: *p* = 0.00; 6–7_2vs. 8–9_2: *p* = 0.00), while only 4–5 years old normally sighted participants showed a similar trend exclusively in condition 2 (interaction between age groups × conditions; RSS = 0.35, iter = 5000, *p* < 2e–16; and Bonferroni: 4–5_2 vs. 6–7_2: *p* = 0.00; 4–5_2 vs. 8–9_2: *p* = 0.00).

**FIGURE 7:**
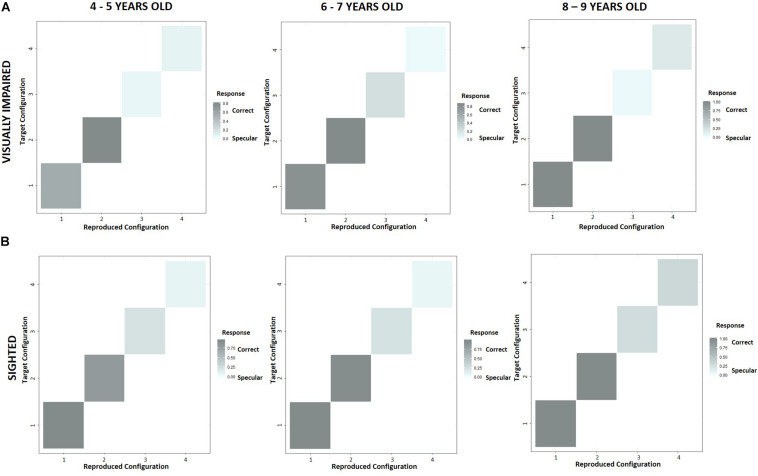
Intra-group performance across age groups in terms of correct and specular responses with confusion matrices. The levels of gray indicate whether participants reproduced a configuration (“Reproduced configuration”, *x*-axis) correctly (dark gray), or specularly (light gray) with respect to the experimenter’s configuration (“Target configuration”, *y*-axis) in a certain condition. **(A)** Visually impaired participants did not improve their performance by relying more on allocentric frames of reference, **(B)** while the number of specular responses given by normally sighted children resulted higher in allocentric (3, 4) than egocentric (1, 2) conditions, gradually reducing with growth.

**FIGURE 8:**
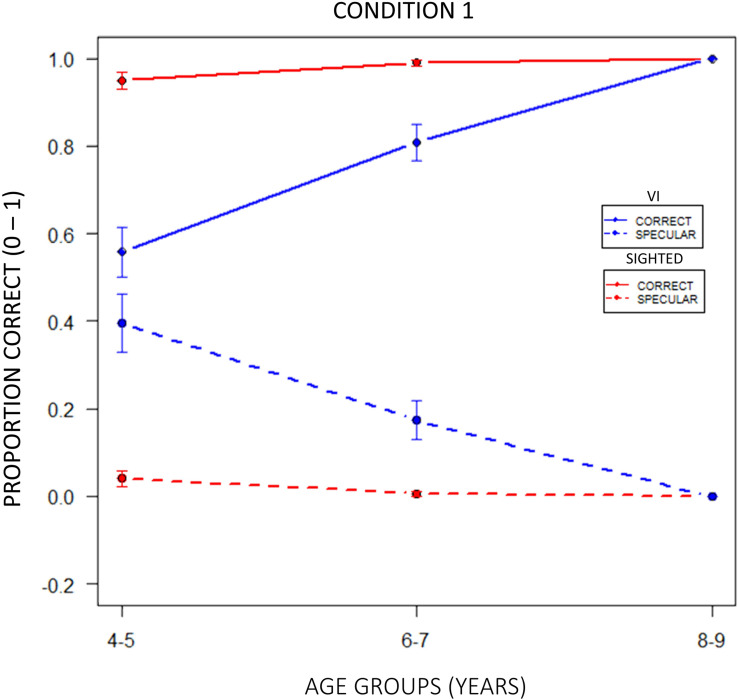
Correct and specular responses in egocentric condition 1. The tendency to reproduce specular configurations was significantly higher in 4–5 years old visually impaired than normally sighted participants (RSS = 0.44, iter = 5000, *p* < 2e–16; and Bonferroni: 45VI_1 vs. 45S_1: *p* = 0.00). This result might suggest that youngest visually impaired children have a significant developmental delay in the consolidation process of egocentric spatial competences.

To evaluate a possible influence of the experimental condition on the reproduction of a configuration, we reported the performance across age groups in terms of correct and specular responses scored by the two experimental groups with the use of confusion matrices (Figure 7). The levels of gray indicate whether participants reproduced a configuration (“Reproduced configuration”, *x*-axis) correctly (dark gray) or specularly (light gray) with respect to the experimenter’s configuration (“Target configuration”, *y*-axis) in a specific condition. As regards normally sighted children (Figure 7B), the number of specular responses resulted higher in allocentric (3, 4) than egocentric (1, 2) conditions, but it gradually reduced with growth. On the contrary, visually impaired participants did not improve their performance by relying more on allocentric frames of reference (Figure 7A). Interestingly, at 4–5 years of age, specular responses given by visually impaired children seemed to be higher than normally sighted peers even in the condition 1, where we expected a similar result based on egocentric cues. Figure 8 confirms that the tendency to reproduce specular configurations in an egocentric condition (1) was significantly higher in 4–5 years old visually impaired than normally sighted participants (inter-groups analysis; interaction between subjects *x* age groups *x* conditions; RSS = 0.44, iter = 5000, *p* < 2e–16; and Bonferroni: 45VI_1 vs. 45S_1: *p* = 0.00). This result suggests a significant developmental delay in the consolidation process of egocentric spatial competencies in youngest visually impaired children.

Overall, our findings suggested that different developmental abilities to localize spatial stimuli by relying on egocentric and allocentric reference frames depend on the amount of visual experience along with childhood.

## Discussion

Several studies indicate that the capability of individuals to integrate egocentric and allocentric frames of reference emerges during the first years of life and typically relies on visual experience. Nonetheless, it is not yet clear when children become able to spontaneously alternate and switch from an egocentric to an allocentric coordinates system depending on task demands and how visual deprivation impacts on this ability. In this work, we tested and verified the hypothesis that children with an atypical visual experience during development (visually impaired) would show a stronger reliance on egocentric frames of reference when a mental update of spatial coordinates was required. In particular, we demonstrated that visually impaired children had more difficulties than typical peers in performing a switching-perspective task. Furthermore, visually impaired children showed a dominance of specular responses (i.e., mirror-like representation of space) at 4–5 years of age in configurations that required an egocentric coordinate system.

Several studies have shown that normally sighted children rely on allocentric cues from an early age. For instance, 3-year-old children can form allocentric representations if provided with environmental cues (Acredolo, 1977, 1978; Acredolo and Evans, 1980; Hermer and Spelke, 1994; Learmonth et al., 2002; Ribordy et al., 2013). They also encode space with egocentric and allocentric coordinates in parallel (Nardini et al., 2006), even if they show a viewpoint-independent perspective only later at five years of age (Nardini et al., 2006). Such findings have been interpreted as the result of cognitive development. Indeed, the first years of life are crucial for the development of executive functions that might play a role in helping children to identify and select the most appropriate spatial strategy according to environmental features (Hermer and Spelke, 1994; Nardini et al., 2008, 2009; Vasilyeva and Lourenco, 2012).

Since vision is crucial for the maturation of spatial cognition (Foulke, 1982; Thinus-Blanc and Gaunet, 1997), the impoverishment of visual feedback can determine impairments in updating spatial coordinates from an egocentric to an allocentric perspective (and vice-versa). Visual impairment can, therefore, produce a developmental delay in spatial planning abilities (Cappagli and Gori, 2016). During development, partial visual deprivation negatively affects the acquisition of spatial competences, resulting in a delay in locomotor and proprioceptive skills (Levtzion-Korach et al., 2000; Bremner et al., 2008). In this work, we found that visually impaired children remained anchored to an egocentric representation of space across ages when they were required to solve the task by using allocentric frames of reference. On the contrary, normally sighted children gradually improved their performance across development, showing an increase in the number of correct responses. This result is in line with previous studies that have reported a deficit of visually impaired children in solving tasks based on the mental rotation of the self (Huttenlocher and Presson, 1973; Millar, 1976; Papadopoulos and Koustriava, 2011; Koustriava and Papadopoulos, 2012) or of the objects (Huttenlocher and Presson, 1973; Penrod and Petrosko, 2003; Papadopoulos and Koustriava, 2011). Recently, it has been hypothesized that the object-centered representation of space cannot be independent of egocentric coordinates (Filimon, 2015). In other words, spatial decisions remained anchored to a purely egocentric spatial reference frame even when spatial locations are referred to external objects. Therefore, it seems that spatial choices are based on a two-steps process. The first step allows to code space in body-centered coordinates, the second step allows relating body-centered to objects-centered coordinates. We can speculate that visually impaired children remained anchored to the first step, being able to code space in egocentric coordinates while not being able to rotate mentally body-centered representation according to the spatial layout. Furthermore, the difficulty in developing allocentric spatial coding skills might be related to different processing of egocentric and allocentric representation at cortical level. Nadel and Hardt (2004) have shown that allocentric and egocentric spatial information are processed in at least partly separate neural networks. Other studies have demonstrated that the activation of the posterior parietal/frontal network and of the posteromedial/medio-temporal cerebral substructures have been reported during egocentric and allocentric spatial coding, respectively (Galati et al., 2010).

Another interesting finding was that visually impaired children manifested a developmental delay in performing the task also from an egocentric point of view. Indeed, in condition 1 (see Figure 2A) they produced more specular than egocentric responses at 4–5 years of age, while normally sighted peers correctly maintained the same perspective (egocentric). At the same age, visually impaired children manifest less specular responses in condition 2, which still required an egocentric perspective, even if two boards lying one above the other were presented (condition 1). In this case, the body midline might play a role in the representation of space based on body coordinates. It has been shown that the body midline can be reliable as a body-centered frame of reference when spatially aligned with the coded object (Millar, 1981, 1985), but not in the case of a body midline-crossing spatial task (Millar and Ittyerah, 1992). Moreover, some works have assumed that egocentric spatial coding may also be centered on the eye (Rock, 1997). According to this egocentric dichotomy, results obtained in condition 1 might suggest that visually impaired children tend to refer more on their body midline at early ages to encode body midline-crossing space. In contrast, they mainly refer to their visual residual in case of body midline-aligned space.

To conclude, we evaluated whether visually impaired children acquire allocentric spatial abilities across development similarly to normally sighted children. We defined a switching-perspective task, in which children were asked to reproduce a visual configuration by changing their position in space thus assuming different spatial reference frames. Our work suggests that an impoverished visual experience during development negatively impacts on the development of allocentric spatial coding and the acquisition of a correct body-center perspective in case of body midline-crossing targets. In order to understand whether vision is required to develop spatial competences, future works should assess whether a complete loss of vision from birth, such in the case of congenital blindness, would produce similar or contrasting results. These findings would favor the development of rehabilitative intervention addressed to children’s needs.

## Data Availability Statement

The datasets generated for this study are available on request to the corresponding author.

## Ethics Statement

The studies involving human participants were reviewed and approved by the Ethics Committee of Pavia Area, Fondazione IRCCS Policlinico San Matteo, Pavia (Italy). Written informed consent to participate in this study was provided by the participants’ legal guardian/next of kin.

## Author Contributions

CM, GC, MG, and SS developed the study concept and design. CM and AL collected the data and CM analyzed the data. CM and GC wrote the manuscript. SS and MG provided critical inputs to review the manuscript.

## Conflict of Interest

The authors declare that the research was conducted in the absence of any commercial or financial relationships that could be construed as a potential conflict of interest.
